# Accurate identification and epidemiological characterization of *Burkholderia cepacia* complex: an update

**DOI:** 10.1186/s12941-019-0306-0

**Published:** 2019-02-04

**Authors:** Naveen Kumar Devanga Ragupathi, Balaji Veeraraghavan

**Affiliations:** Department of Clinical Microbiology, Christian Medical College, Vellore, Tamil Nadu 632004 India

**Keywords:** *Burkholderia cepacia* complex, Cystic fibrosis, Hospital acquired infections, *rec*A, *his*A, *rps*U

## Abstract

Bacteria belonging to the *Burkholderia cepacia* complex (Bcc) are among the most important pathogens isolated from cystic fibrosis (CF) patients and in hospital acquired infections (HAI). Accurate identification of Bcc is questionable by conventional biochemical methods. Clonal typing of *Burkholderia* is also limited due to the problem with identification. Phenotypic identification methods such as VITEK2, protein signature identification methods like VITEK MS, Bruker Biotyper, and molecular targets such as 16S rRNA, *rec*A, *his*A and *rps*U were reported with varying level of discrimination to identify Bcc. *rps*U and/or 16S rRNA sequencing, VITEK2, VITEK MS and Bruker Biotyper could discriminate between *Burkholderia* spp. and non-*Burkholderia spp*. Whereas, *Bcc* complex level identification can be given by VITEK MS, Bruker Biotyper, and 16S rRNA/*rps*U/*rec*A/*his*A sequencing. For species level identification within Bcc *his*A or *rec*A sequencing are reliable. Identification of Bcc is indispensable in CF patients and HAI to ensure appropriate antimicrobial therapy.

## Background

*Burkholderia cepacia* is generally an environmental plant pathogen, second common to ESKAPE pathogens in humans. They were uncovered due to natural calamities and construction activities due to lack of infrastructure in developing nations. Differentiation of species within the *B. cepacia* complex (Bcc) can be particularly problematic, even with an extended panel of biochemical tests [1], as they are phenotypically very similar and most commercial bacterial identification systems cannot reliably distinguish between them. Further, reliable differentiation of these species from other related taxa, such as *Ralstonia, Cupriavidus, Pandoraea, Achromobacter, Brevundimonas, Comamonas* and *Delftia* species is challenging.

In most cases from developing nations, BCC has been misidentified as non-fermentative Gram-negative bacilli (NFGNB) especially *Pseudomonas* spp. [2, 3]. Due to which, reports on Bcc infections are rare in India [4].

### Selection of literature for review

The articles were searched using PubMed (https://www.ncbi.nlm.nih.gov/pubmed/) and Google Scholar. Multiple keywords were used for the literature search in combination or in alone. Some of the important keywords used for literature search were *Burkholderia cepacia* complex (Bcc), hospital acquired infections, phenotypic identification of Bcc, molecular identification of Bcc. *B. cepacia*, *B. cenocepacia*, phoenix, VITEK2, VITEK MS, Bruker biotyper, recA, hisA, rspU, 16S rRNA and WGS of *B. cepacia*/Bcc.

## Main text

### Classification of *Burkholderia cepacia complex*

#### Basic taxonomy

Walter H. Burkholder described a phytopathogenic bacterium causing onion rot in New York State in the mid-1940s and named the species ‘cepacia’ [5]. This was initially known as *Pseudomonas cepacia*, later in 1992 included in the Betaproteobacteria class, with Burkholderiales order and *Burkholderiaceae* family as *Burkholderia cepacia* [6]. *Burkholderia* includes former rRNA group II pseudomonads (*Pseudomonas gladioli*, *Pseudomonas mallei*, *Pseudomonas pseudomallei*, and *Pseudomonas caryophylli*), except *Pseudomonas pickettii* and *Pseudomonas solanacearum,* which were later grouped under the genus *Ralstonia* [7]. *Burkholderia* species were known as plant pathogens and soil bacteria, except *B. mallei* and *B. pseudomallei,* which are humans and animal pathogens [8].

The genus now includes 22 validly described species: *B. cepacia* (the type species), *Burkholderia caryophylli*, *Burkholderia mallei*, *Burkholderia pseudomallei*, *Burkholderia gladioli*, *Burkholderia plantarii*, *Burkholderia glumae*, *Burkholderia vietnamiensis*, *Burkholderia andropogonis*, *Burkholderia multivorans*, *Burkholderia glathei*, *Burkholderia pyrrocinia*, *Burkholderia thailandensis*, *Burkholderia graminis*, *Burkholderia phenazinium*, *Burkholderia caribensis*, *Burkholderia kururiensis*, *Burkholderia ubonensis*, *Burkholderia caledonica*, *Burkholderia fungorum*, *Burkholderia stabilis*, and *Burkholderia ambifaria* [9].

Since the mid-1990s, heterogeneity was noted among the *B. cepacia* strains isolated from different ecological niches. This caused problems in accurate identification of *B. cepacia* isolates, and evaluation of the techniques used showed that they were either not very sensitive, not very specific, or neither sensitive nor specific [10–13].

Further, Vandamme et al. [14] evaluated a polyphasic taxonomic approach to demonstrate that presumed “*B. cepacia*” from CF patients and other sources were different and belonged to five distinct genomovars (phenotypically similar genomic species). This includes, *B. cepacia* genomovar I, *B. multivorans* genomovar II, genomovar III, *B. stabilis* genomovar IV and *B. vietnamiensis* genomovar V. Initially these five genomic species were collectively referred to as the *B. cepacia* complex (Bcc). Subsequent polyphasic taxonomic studies identified genomovar VI and *B. ambifaria* genomovars VII which added to Bcc [15, 16]. In addition, *B. pyrrocinia* was added to Bcc [17].

*Ralstonia, Cupriavidus, Pandoraea, Achromobacter, Brevundimonas, Comamonas* and *Delftia* are the most common genus those are closely related to the *Burkholderia* and cause problems in accurate identification of Bcc. These are hitherto referred as non-*Burkholderia spp*. in this manuscript. Similarly, *Burkholderia* spp. (*B. humptydooensis* and *B. pseudomallei* complex) which interferes in correct identification of Bcc are referred as non-Bcc.

#### Molecular phylogeny

Previously, different species within the *B. cepacia* complex had shown to have DNA–DNA hybridisation values between 30 and 60%, while strains of same species showed values > 70%. Whereas, values obtained with non-Bcc Burkholderia were below 30% [14–16, 18–20]. The DNA relatedness is rated as high (> 70%) in strains of same species, low (30–60%) but significant below the species level, and non-significant (< 30%).

Coenye et al. [15], has compared the 16S rDNA sequences of *B. cepacia* complex and related species, where, the similarities of strains within *B. cepacia* complex were higher (> 97.7%) compared to other *Burkholderia* species (< 97.0%).

#### Biochemical reactions

Different media composition were in use for years to selectively isolate *B. cepacia* complex from samples of CF patients. This includes, *P. cepacia* medium (PC agar) (300 U of polymyxin B/ml and 100 µg of ticarcilline/ml) [21]; Oxidation-fermentation agar with lactose and polymyxin B (OFPBL agar) (300 U of polymyxin B/ml and 0.2 U of bacitracin/ml) [22], and *B. cepacia* selective agar (BCSA) (1% lactose and 1% sucrose in an enriched base of casein and yeast extract with 600 U of polymyxin B/ml, 10 µg of gentamicin/ml, and 2.5 µg of vancomycin/ml) [23]. BCSA was proven effective than the other two in recovering *B. cepacia* complex from CF respiratory specimens by inhibiting growth of other organisms [24]. Though, *B. gladioli* and *Ralstonia* spp. are exceptions which could grow on BCSA. On isolation, few biochemical reactions used to differentiate *B. cepacia* complex, *B. gladioli*, *Pandoraea* spp., *R. pickettii*, *A. xylosoxidans*, and *S. maltophilia* are enlisted in Table 1.

**Table 1:** Biochemical characteristics to differentiate *B. cepacia* complex, *B. gladioli*, *Pandoraea* spp., *R. pickettii*, *A. xylosoxidans*, and *S. maltophilia*

	*B. cepacia*							*B. gladioli*	*Pandoraea species*	*R. pickettii*	*A. xylosoxidans*	*S. maltophilia*
	Genomovar I	Genomovar II	Genomovar III	Genomovar IV	Genomovar V	Genomovar VI	Genomovar VII					
Oxidase	+	+	+	+	+	+	+	‒	v	+	+	‒
Oxidation of:												
Sucrose	v	‒	v	‒	+	‒	+	‒	‒	‒	‒	v
Adonitol	v	+	v	v	–	+	+	+	–	–	–	–
Lactose	v	+	v	+	+	+	+	‒	‒	‒	‒	+
Lysine decarboxylase	+	v	+	+	+	‒	+	‒	‒	‒	‒	+
Ornithinie decarboxylase	v	‒	v	+	‒	‒	‒	‒	‒	‒	‒	‒
Gelatine liquefaction	v	‒	v	+	‒	‒	+	v	‒	‒	‒	+
Aesculine hydrolysis	v	‒	v	‒	‒	‒	v	v	‒	v	‒	+
β-Galactosidase activity	+	+	+	‒	+	+	+	+	‒	‒	‒	+
Growth at 42 °C	v	+	v	‒	+	+	v	‒	v	v	NK	v
β-Hemolysis	‒	‒	‒	‒	v	‒	v	‒	‒	‒	NK	NK

Recent developments had led to invention of automated/commercial test systems for pathogen identification. However, there are several reports pertaining to inability of these commercial systems to identify or differentiate *B. cepacia* complex isolates from other *Burkholderia* spp. [25].

### Bcc in cystic fibrosis

Most often, cases with fulminating pneumonic infection along with fever and respiratory failure, occasional association with septicaemia, is known as “*cepacia* syndrome” [26]. The overwhelming *B. cepacia* complex infections in cystic fibrosis patients have prompted an unusual number of studies and variety of data. *B. cepacia* was also frequently encountered in nosocomial outbreaks due to contaminated disinfectants, nebulizer solutions, mouth wash, medical devices and intravenous solutions due to contamination of lipid emulsion stoppers [27]. Though, *B. multivorans* and *B. cenocepacia* were reported predominant amongst CF patients than non-CF patients as reported from United States, Canada, Italy and Australia [16, 28, 29].

### Problems in accurate identification of *Burkholderia* spp.

Phenotypic tests either manual or automated commercial systems were in use to identify Bcc in routine clinical laboratories. Though, species level identification is not achieved due to high similarity of biochemical results between species. Automated identification systems including Phoenix, VITEK 2, VITEK MS and Bruker identifies Bcc, non-Bcc and non-*Burkholderia* spp. at different specificities (Table 2) [30–33].

**Table 2:** Biochemical and molecular identification of *Burkholderia cepacia* complex in hospital acquired infections

	Methods	Target	Identification	Remarks
Phenotypic methods	Conventional biochemical method	Catalase, Gluconate, Malate, Phenylacetate, leucine arylamidase activity	Overlapping biochemical profiles for Bcc, *Ralstonia* spp. and *Pandoraea* spp.	Bcc and non-Bcc cannot be distinguished
	Phoenix	Biochemicals—automated	Cannot identify *Ralstonia pickettii*	Misidentification rate for Bcc is 23%
	VITEK 2	Biochemicals—automated	Can identify *Ralstonia pickettii* (83%)	Misidentification rate for Bcc is 12%
Protein signature	VITEK MS	Mass spectrogram of the protein	Genus level identification of *B. cepacia*—55–63% *R. pickettii* identification—85–100% *Pandoraea* spp.—87%	Species within Bcc cannot be distinguished
	Bruker Biotyper	Mass spectrogram of the protein	Agreement between Bruker Biotyper and *rec*A sequencing Genus level—100% Species level—76.9% *B. cenocepacia*—95.8–100% *B. multivorans*—78.5% *B. contaminans*—0% *B. vietnamiensis*—100% *B. cepacia*—30–33.3%	Can identify and discriminate Bcc from non-*Burkholderia* spp. Few species within Bcc cannot be distinguished
Molecular targets	*rec*A	DNA recombinase enzyme for DNA repair	Promising for differentiation of *Burkholderia* species including Bcc	Non-Bcc cannot be distinguished
	*his*A	Encodes for an enzyme involved in histidine biosynthesis	Could discriminate among the Bcc species	Non-Bcc cannot be distinguished
	*rsp*U	Coding for a ribosomal protein S21	Burkholderia spp. and non-*Burkholderia* spp. can be distinguished	Species within Bcc cannot be distinguished
	16S rRNA	Component of the 30S small subunit of a prokaryotic ribosome	*Burkholderia* spp. and non-*Burkholderia* spp. can be distinguished	Unacceptable for discrimination of Bcc intra-species

There is considerable interest in recent days on the reliability of MALDI-TOF MS for accurate bacterial identification. It is based on the spectral analysis of bacterial proteins, mainly ribosomal proteins, ionized by laser irradiation of the bacterial cell. Fehlberg et al. [25] has evaluated the performance of MALDI-TOF MS for species identification of Bcc clinical isolates in comparison to *rec*A sequencing. MALDI-TOF MS results were 100% in concordant with *rec*A sequencing for genus level identification (*n* = 91), while 76.9% (*n* = 70) concordance was seen for species level identification. Another study by Gautam et al. [34], has compared MALDI-TOF MS with an expanded MLST and *rec*A sequencing for Bcc identification. MALDI-TOF MS exhibited 100% concordance for genus identification and 82% for species level identification.

The accuracy in the identification and differentiation of *Burkholderia* spp. in clinical specimens with the close neighbours *Pandoraea*, *Cupriavidus* and *Ralstonia* is essential for the treatment of patients. These three are the most prevalent genera identified outside *Burkholderia* genus. Most of the time these are phenotypically misidentified as Bcc.

*Pandoraea* species have been reported from both cystic fibrosis (CF) and non-CF patients. The invasive potential of this genus can be understood through various reported cases of *Pandoraea* bacteraemia caused by *P. pnomenusa*, *P. apista*, *P. pulmonicola* and *P. sputorum* [35–40], where identification was a major setback when conventional biochemical methods were used.

The *Ralstonia* genus includes *R. pickettii* and *R. solanacearum* (formerly *Burkholderia pickettii* and *B. solanacearum*), *R. insidiosa*, and *R. mannitolilytica*, where *R. pickettii* is still regarded as the main pathogenic species [41]. Though *R. pickettii*, is considered with minor clinical significance, many instances of infections are reported in the literature. Due to high similarity between *R. pickettii* and Bcc, many of the Bcc cases might have been misidentified which are actually *R. pickettii* [42]. Contaminated solutions including water for injection, saline solutions made with purified water, and sterile drug solutions were regarded as the cause of *R. pickettii* infections in many of the cases. Major conditions associated with *R. pickettii* infection are bacteraemia/septicaemia and respiratory infections/pneumonia [41, 43, 44].

Very often, *Ralstonia* and *Pandaroeae* are misidentified as Bcc. These genus are very closely related to *Burkholderia* spp., such that they cannot be distinguished by standard biochemical method [35]. The species include Bcc (*B.* *cepacia*, *B.* *multivorans*, *B.* *cenocepacia*, *B.* *vietnamiensis*, *B.* *stabilis*, *B.* *ambifaria*, *B.* *dolosa*, *B.* *anthina*, *B.* *pyrrocinia* and *B.* *ubonensis*), *B. humptydooensis*, *Cupriavidus* spp., *Pandoraea* spp., and *B. pseudomallei*.

**Fig. 1:**
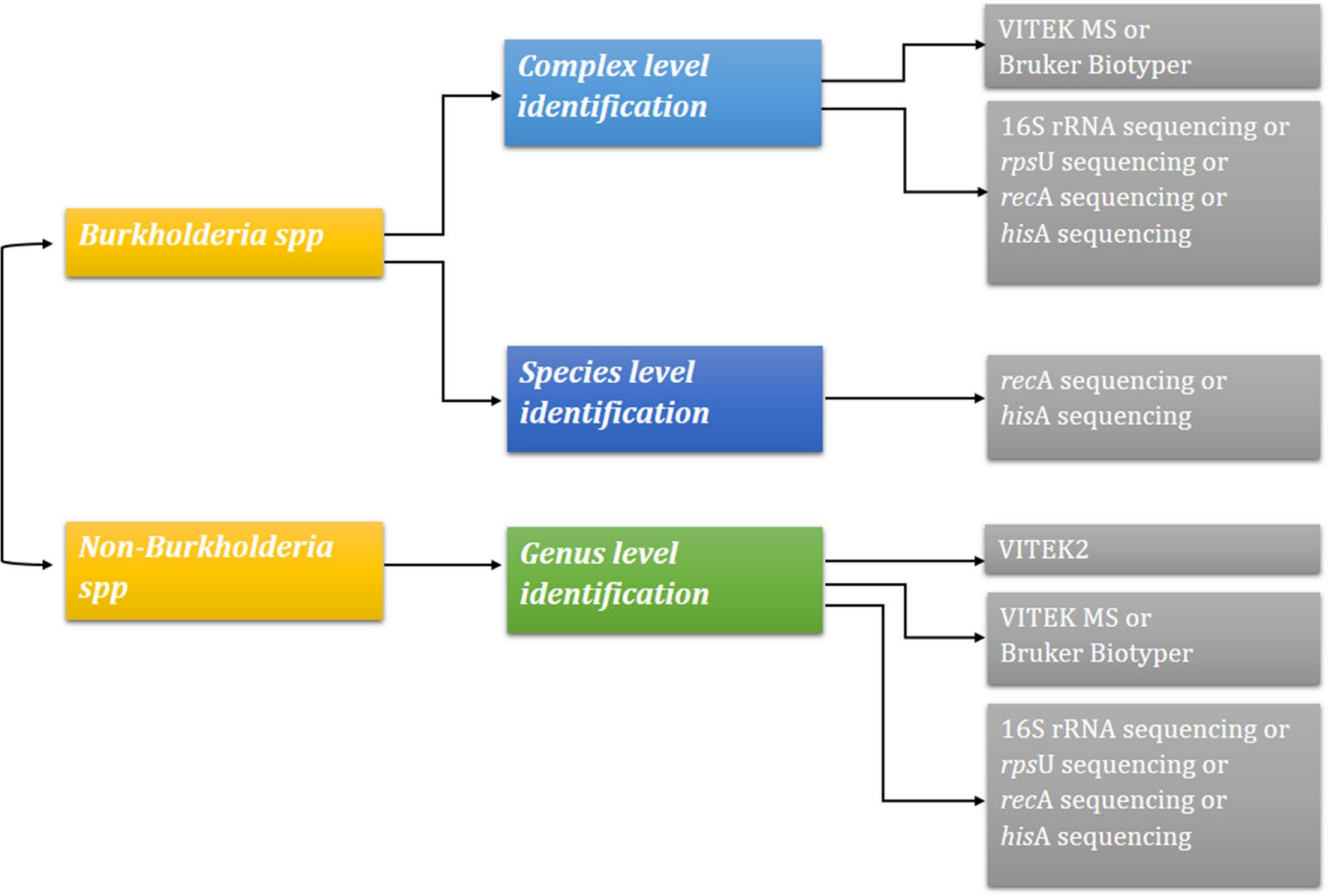
Algorithm depicting the methods for accurate identification of *Burkholderia* at genus level (near neighbouring genus), cepacia complex level (Bcc) and species level (within Bcc)

Bcc and non-*Burkholderia* spp. could not be distinguished by conventional biochemical methods. Due to problem with identification, clonal typing of *Burkholderia* is questionable. Molecular targets such as 16S rRNA, *rec*A, *his*A and *rps*U were reported to increase the discrimination of *Bcc*. Representation of various techniques and its ability to accurately identify Bcc is given in Fig. 1.

### Need for molecular identification of Bcc

Isolates from CF patients with persistent pathogenic colonization often lose their characteristic phenotypes or growth conditions which leads to difficulty in accurate identification of Bcc. To overcome this, molecular identification is required to distinguish species within Bcc and from the related genus/species. Though molecular targets for identification are not reliable when used individually, a multi-target approach is essential to improve the identification of Bcc and non-Bcc organisms. Some of the reported molecular targets are *his*A, *rps*U, *rec*A and 16S rRNA. Discriminating ability on genus/complex/species level using these targets were listed in Table 2.

### *hisA* and *rpsU* gene sequencing

Sequencing of *his*A gene, encodes for an enzyme involved in histidine biosynthesis was reported to distinguish species within Bcc [45]. Neighbour-joining method analysis of 134 Bcc organisms revealed high degree of sequence similarity between strains of same species. Meanwhile, each species was clearly separated from each other. The *his*A based analysis separated 17 Bcc species in different clusters (including 4 lineage divisions of *B. cenocepacia*) with high bootstrap values (> 75%) [45]. *Burkholderia* strains used for *his*A based analysis were previously identified using a polyphasic taxonomy or *rec*A sequencing [46, 47].

Similarly, rpsU was recognized to identify different species among the Burkholderia genus [48]. Frickmann et al. [48] has employed rpsU sequencing method to compare Burkholderia strains of known identity from ATCC (American Type Culture Collection, Manassas, Virginia, USA), DSMZ (German Collection of Microorganisms and Cell Cultures, Braunschweig, Germany), JCM (Japan Collection of Microorganisms, Tsukuba, Ibaraki Prefecture, Japan), BCCM/LMG (Bacteria Collection, Ghent, Belgium), and NCTC (National Collection of Type Cultures, Porton Down, UK). Also few clinical strains were included in the analysis for comparison, after ensuring their identity using *recA* sequencing. rpsU sequences formed four clusters including *B. plantarii*, *B. glumae*, *B. cocovenenans* and *B. gladioli* in cluster I, the Burkholderia pseudomallei complex (*B. mallei*, *B. pseudomallei*, and *B. thailandensis*) in cluster II, *B. caryophylli*, *B. multivorans*, *P. norimbergensis*, *B. ubonensis*, *B. stabilis*, *B. cenocepacia*, *B. cepacia*, *B. pyrrocinia*, *B. ambifaria*, *B. anthina*, *B. vietnamiensis* and *B. dolosa* in cluster III, and *B. sacchari*, *B. graminis*, *B. fungorum*, *B. phytofirmans*, *B. xenovorans*, *B. phenoliruptrix*, *B. phenazinium*, *B. caribensis*, *B. hospita* and *B. phymatum* in cluster IV. *B. glathei*, *B. caledonica* and *B. kururensis* were observed as outliers.

Moreover, the *rps*U sequence homology for *Burkholderia* and *Pandorea* was > 86%. Most of the clinical pathogens of Bcc belongs to cluster III of *rps*U sequencing, where *B. caryophylli*, *B. multivorans*, and *P. norimbergensis* had identical sequences and *B. cenocepacia* clustered with *B. cepacia*. Limitation of *rps*U sequencing is it could not reliably discriminate *Burkholderia* spp. at the species level as single target.

### *recA* gene sequencing

*rec*A is another well-known target promising for differentiation of *Burkholderia* species [46]. *rec*A can differentiate the following 19 species of *Burkholderia* namely, *B. pseudomallei*, *B. mallei*, *B. thailandensis*, *B. humptydooensis*, *B. oklahomensis*, *B. oklahomensis*-like, *B. ubonensis*, *B. ambifaria*, *B. multivorans*, *B. vietnamiensis*, *B. fungorum*, *B. glumae*, *B. cepacia*, *B. xenovorans*, *B. dolosa*, *B. gladioli* and Bcc [49]. However, non-*Burkholderia* spp. cannot be distinguished by *rec*A sequencing. *Burkholderia* strains used for evaluation of *rec*A sequencing were characterised using whole-cell protein profile analysis and a polyphasic approach [47, 50].

## 16S rRNA sequencing

16S rRNA sequencing is one important option for differentiating non-*Burkholderia* spp. from *Burkholderia* spp. [49]. The 16S rRNA similarity between *Burkholderia* spp. and non-*Burkholderia* spp. is given in Table 3. In a study from environmental sample, the different species of *Burkholderia* including non-*Burkholderia* spp. were found in the same consortium suggesting that same environmental niche hosts the sharing of genes through lateral gene transfer [51]. Due to these challenges in identification, the clonality of these species is also difficult to investigate, as the MLST housekeeping genes are species specific. As of now, MLST database is available only for Bcc and *B. pseudomallei*. HAI outbreaks caused due to non-*Burkholderia* spp. could not be typed.

**Table 3:** Similarity of 16S rRNA sequences between *Burkholderia* and non-*Burkholderia* spp.

% Similarity of 16S rRNA	*Pandoraea*	*B. stabilis*	*B. cenocepacia*	*B. cepacia*	*B. multivorans*	*B. pyrrocinia*	*B. vietnamiensis*	*B. ambifaria*	*B. anthina*	*B. humptydooensis*	*B. pseudomallei*	*B. mallei*	*B. cupriavidus*	*Ralstonia*
1: *Pandoraea*	100													
2: *B. stabilis*	95.16	100												
3: *B. cenocepacia*	94.02	98.32	100											
4: *B. cepacia*	95.1	98.38	98.32	100										
5: *B. multivorans*	95.28	98.46	97.43	99.79	100									
6: *B. pyrrocinia*	95.72	99.09	97.91	99.02	99.1	100								
7: *B. vietnamiensis*	95.48	98.67	97.92	99.44	99.4	99.17	100							
8: *B. ambifaria*	95.76	99.23	97.92	99.02	99.12	99.45	99.32	100						
9: *B. anthina*	95.85	99.33	99.7	99.11	99.19	99.41	99.56	99.7	100					
10: *B. humptydooensis*	94.86	97.54	96.38	97.55	97.59	97.86	98.02	98.23	98.22	100				
11: *B. pseudomallei*	95.05	97.47	96.52	97.83	97.83	97.99	98.25	98.29	98.08	98.92	100			
12: *B. mallei*	95.07	97.4	96.45	97.76	97.79	97.93	98.22	98.23	98	98.89	99.87	100		
13: *B. cupriavidus*	91.5	90.99	91.29	91.29	91.21	91.14	91.14	91.21	91.56	91.49	91.63	91.63	100	
14: *Ralstonia*	92.39	90.53	89.58	90.97	91.03	91.16	91.37	91.23	91.49	91.56	91.63	91.63	94.66	100

### Whole genome sequences of *B. cepacia*

Due to recent developments in the molecular genetics of bacteria, usage of whole genome sequences (WGS) of the bacterial pathogens is gaining interest among clinical microbiologists. The WGS data helps in typing of the pathogens and in identifying the evolutionary pattern of the organism based on whole genome single nucleotide polymorphisms (SNPs). Till date 102 genome sequences have been deposited in NCBI for *B. cepacia*. This includes complete genomes and shotgun genome sequences. Further baseline data on *B. cepacia* WGS will help to identify region specific clones, which will be handy in identifying an outbreak situation.

### Antimicrobial resistance mechanisms and therapy of Bcc

The mechanisms of antibiotic resistance of Bcc species have been intensively studied. Major resistance mechanism in Bcc is due to efflux pump overexpression mostly by members of the resistance-nodulation-division (RND) family [52–54]. *B. cenocepacia* strain J2315 was reported to encode 16 RND efflux systems [55, 56].

Ceftazidime and other extended-spectrum cephalosporins are the reliable treatment options for Bcc due to intrinsic resistance to many other classes of antimicrobials. Bcc were reported with class A β-lactamases conferring resistance to β-lactam antibiotics such as ceftazidime. This was first described in *B. cepacia* as the PenA-PenR system [57], which were later known as PenB and PenR (AmpR) [58]. Due to their complex role in activation of *penB* and *ampC* targets in the presence of an antibiotic susceptibilities to ceftazidime, cefotaxime, and meropenem were greatly reduced [58, 59].

Class A PenA β-lactamase was also reported in *B. cenocepacia* which is located on chromosome 2 and their genetic environment are similar to that of *B. pseudomallei* [60]. However, the *B. cenocepacia* enzyme has not yet been shown to be involved in β-lactam resistance. In a study by Hwang and Kim [58], a *B. cenocepacia* strain J2315 PenB β-lactamase had shown a Ser72Tyr substitution, due to which *B. cenocepacia* has intrinsic clavulanate resistance [58].

In addition, *B. multivorans* was reported to have a PenA enzyme (Bmul_3689 in *B. multivorans* ATCC 17616), that is closely related to PenB reported in Bcc [58, 61]. This PenB is also similar to KPC-2 a significant carbapenemase [62]. However, the *B. multivorans* enzyme is an inhibitor-resistant carbapenemase, unlike in *B. pseudomallei* which is an extended spectrum β-lactamase. Though the active role of PenA in clinical *B. multivorans* is it yet established. There were also reports on difference in efflux pump and outer membrane protein mediated resistance especially for colistin in Bcc and *B. pseudomallei* complex (Bpc) [63]. Due to these multiple difference in their resistance mechanisms, it is imperative to accurately identify Bcc from other *Burkholderia spp* for appropriate therapy.

The choice for antimicrobial therapy is usually chosen based on in vitro susceptibility, while duration of therapy be based upon clinical and microbiologic response. Use of combination regimen is commonly reported for Bcc [64]. However, it is still uncertain as the evidences were mostly limited to in vitro studies or small clinical experiences. For serious infection with susceptible strains, a two-drug combination of parenteral trimethoprim-sulfamethoxazole (5 mg/kg trimethoprim component every 6–12 h) plus a β-lactam (e.g., ceftazidime, piperacillin, meropenem) or a fluoroquinolone should be utilized [65]. For serious infection with trimethoprim-sulfamethoxazole-resistant strains or sulfa drug allergy, combination therapy guided by in vitro susceptibility results should be administered [66]. In a study by Blumer et al. [67], in 102 CF patients, meropenem/tobramycin and ceftazidime/tobramycin improved clinical status and reduced bacterial burden in 96 and 92% of treated patients, respectively.

Bonacorsi et al. had proven enhanced bactericidal activity of ciprofloxacin in combination with other agents [68]. Further, triple antimicrobial combination based on meropenem was suggested useful than double or single agents [69].

Macrolides in combination with other antimicrobials had shown moderate synergism [70], while specific combinations including fosfomycin/tobramycin exhibited poor activity against Bcc [71].

## Conclusions

Conventional phenotypic methods could not discriminate *Bcc* and related genus, as there is an overlap in the biochemical characteristics. A single molecular target for differentiation of Bcc from non-Bcc and non-*Burkholderia* spp. is not reliable, while two or more molecular targets significantly improves the species level discrimination in Bcc. *rps*U and/or 16S rRNA sequencing, VITEK2, VITEK MS and Bruker Biotyper could discriminate between *Burkholderia* spp. and non-*Burkholderia spp*. Whereas, *Bcc* complex level identification can be given by VITEK MS, Bruker Biotyper, 16S rRNA/*rps*U/*rec*A/*his*A sequencing. For species level identification within Bcc *his*A or *rec*A sequencing are reliable. Recent advancements in genome sequencing using SNP phylogeny might help to accurately identify the clone of Bcc from non-Bcc and non-*Burkholderia spp*. Such identification is necessary to help in timely diagnosis of hospital acquired infections and to provide appropriate antimicrobial therapy.
